# FUSE binding protein FUBP3 is a potent regulator in Japanese encephalitis virus infection

**DOI:** 10.1186/s12985-021-01697-8

**Published:** 2021-11-18

**Authors:** Peng Xu, Wei Tong, Young-Mao Chen

**Affiliations:** 1 Xiangyang No.1 People’s HospitalHubei University of Medicine, Xiangyang, Hubei Province China; 2 Department of Clinical Laboratory, Xiangyang No.1 People’s Hospital, Hubei University of Medicine, Xiangyang, China; 3 Bachelor Degree Program in Marine Biotechnology, College of Life Sciences, National Taiwan Ocean University, Keelung, 20224 Taiwan; 4 Center of Excellence for the Oceans and Matsu Marine Research Center, National Taiwan Ocean University, Keelung, 20224 Taiwan

**Keywords:** Japanese encephalitis virus, Far upstream element-binding protein 3, Untranslated region, Viral replication

## Abstract

**Background:**

The JEV genome is a positive-sense RNA with a highly structured capped 5′UTR, 3′UTR and a large open reading frame. 3′UTR is the untranslated region of flavivirus and has various important functions during viral replication, such as translation, replication and encapsidation. During viral replication, the 3′UTR interacts with viral proteins and host proteins and is required for viral RNA replication and translocation.

**Methods:**

The expression level of FUBP3 was knocked down by siRNA and Flag-tagged FUBP3 overexpression plasmid was constructed for overexpression. BHK-21 cells were cultured and infected with JEV to investigate the functional role of FUBP3 in the viral infection cycle. Subcellular localization of FUBP3 and viral replication complexes was observed by dual immunofluorescence staining.

**Results:**

Four host proteins were specifically associated with the 3′UTR of JEV, and FUBP3 was selected to further investigate its potential functional role in the JEV infection cycle. Knockdown of FUBP3 protein resulted in a significant decrease in JEV viral titer, whereas ectopic overexpression of FUBP3 resulted in increased JE viral infectivity. In cells stably knocked down for FUBP3 and then infected with JEV, we found almost no detectable viral NS5 protein. In contrast, when cells stably knocking-down of FUBP3 overexpressed FUBP3, we found a significant increase in viral RNA production over time compared to controls. We also demonstrated that FUBP3 re-localized in the cytoplasm after infection with JEV and co-localized with viral proteins. Exogenous overexpression of FUBP3 was also shown to be located in the JE replication complex and to assist viral replication after JEV infection.

**Conclusions:**

The overall results suggest that FUBP3 regulates RNA replication of JEV and promotes subsequent viral translation and viral particle production.

## Introduction

Japanese encephalitis (JE) is an infectious disease caused by a Japanese encephalitis virus (JEV) that infects the central nervous system of humans. Transmission of Japanese encephalitis virus is primarily carried by mosquitoes as vectors and can be transmitted between humans, birds, pigs, bats, and horses, as well as other animals. The incubation period of Japanese encephalitis virus is 4–14 days, and clinical symptoms include fever, headache, vomiting, loss of appetite, and mental disturbance; in severe cases, fever, headache, vomiting, and neurological symptoms appear suddenly, while neurological symptoms include seizures, tremors, photophobia, and limb disorders, and the most severe cases can lead to coma or death [1].

The JEV genome is a single-stranded and positive-sense RNA with a genome size of 10,976 nucleotides. JEV genomic RNA consists of a highly structured capped 5′UTR, a 3′UTR and a large open reading frame (ORF) [2, 3]. When JEV enters the host cell, it releases viral RNA and translates one polyprotein, which is then cleaved by viral NS2B/3 protease and host proteases to obtain the viral structural and nonstructural proteins. Structural proteins are divided into outer capsid proteins, membrane protein precursors and an envelope protein; non-structural proteins are divided into NS1, NS2A, NS2B, NS3, NS4A, NS4B, NS5, which play important roles in viral infection [2, 4–6].

The 3′UTR is the untranslated region of flaviviruses and has various important functions during viral replication, such as translation, replication and encapsidation [7, 8]. This 3′UTR contains several functional motifs, including conserved sequences (CS motifs), cyclization motifs, pseudoknot structure and 3′-stemloop (3′-SL) motif. Past studies have identified two pairs of cyclization motifs in which the 5′ and 3′ conserved sequences complement to each other and may form the cyclization structure [9]. The upstream of the AUG codon (5′UAR) is located in the second pair of cyclization motifs, which is complementary to the sequence located in the 3′UTR (3′UAR). The replication of DENV and WNV confirmed the necessity of the existence of the UAR cyclization motif [10]. In addition to this, another element located downstream of the AUG (designated as 5′DAR) has been shown to be involved in the cyclization of the genome during the replication of DENV [11]. It has also been reported that the 3′UTR of JEV is a structural domain of six components [12]. Two of these 3′-proximal structural domains are sufficient for the replication of viral RNA [13]. In addition to RNA–RNA interactions within the viral genome, the 3′UTR of JEV interacts with viral proteins and host proteins that are required for viral RNA replication and translocation [14].

The far upstream element-binding proteins (FUBP) comprise a three-gene family whose physiological functions are primarily single-stranded nucleic acid binding proteins. FBP (encoded by FUBP1), the 2 other proteins, including FUBP2 encoded by KHSRP and FBP3 encoded by FUBP3 [15]. Several studies have shown that FUBPs interact with many DNA or RNA targets in the cells and are further involved in various biological processes, including RNA transport, translational regulation, mRNA degradation, and transcription [16–18]. The structure of FUBPs includes a central structural domain consisting of four regularly spaced K homology (KH) patterns that recognize similar sequences in single-stranded DNA or RNA targets [19]. As confirmed by various in vitro and tissue culture experiments, FUBPs bind to complex DNA and RNA targets. Many neoplastic diseases are closely associated with dysregulation of FUBPs. Although high levels of FUBP expression are associated with poor tumor growth and prognosis in hepatocellular carcinoma, non-small cell lung cancers and gliomas, FUBP expression accounts for a significant proportion of oligodendrogliomas, where it is a tumor suppressor. FUBP1 is widely expressed in a number of tissues and cells with a distinct spatial–temporal profile, and it affects a number of cellular processes through the effects of transcription, mRNA stability, and translation, including differentiation, cell proliferation, apoptosis or cell death [20–24]. Aberrant expression of this gene has been found in malignant tissues, and the gene is important for neural system and lung development. The binding of this protein to viral RNA is thought to play a role in several viral diseases, including hepatitis C and hand–foot–mouth disease. FUBP3 is a single-stranded DNA-binding protein that recognizes FUSE. The gene encoding FUBP3 is located on chromosomes 9q34.1 [25, 26]. Studies have shown that FUBP3 provides substantial support for the proposed role of FUBP1 and FUBP3 as activators of c-myc in vivo [27]. In addition, FUBP3 was recently reported to interact with the 5′ untranslated region of enterovirus 71 (EV-A71) to regulate its replication in differentiated neuronal cells [28].

In the present study, we identified four host proteins specificities associated with the JEV 3′UTR, and FUBP3 and FUBP1 were among them. Since FUBP1 has been reported to negatively regulate the infection cycle of JEV [29], this study focused on the possible role of FUBP3 in the JEV infection cycle.

## Methods

### Cell culture and viruses

We used RPMI 1640 medium (Gibco-Invitrogen, Carlsbad, CA, USA), cultured Baby Hamster Kidney-21 (BHK-21) cells supplemented with 2% fetal bovine serum (FBS) (Gibco-BRL, Carlsbad, CA, USA) and 24 mM sodium bicarbonate (Sigma, St. Louis, USA), and maintained at 37 °C in an atmosphere of 5% CO_2_. Virus stocks were obtained from Dr. WJ Chen and referred to as T1P1 strain (accession number: AF254453). For viral propagation, we infected the BHK-21 cells at a multiplicity of infection (MOI) of 2. BHK-21 cells infected with JEV were then cultured for 2 days before being harvested for further experiments.

### RNA pull-down experiments and LC–MS/MS analysis

The construction of plasmid pJEV-3′UTR was previously described [30]. The 3′UTR fragment flanked by BamHI sites was excised from the pUC19 vector, and in vitro RNA transcripts were obtained using the HiScribeTM T7 in vitro transcription kit (New England bioLabs). Biotinylated RNA was synthesized in a mixture containing NTP, bio-labeled UTP, reaction solution and other reagents according to the kit protocol. The non-biotinylated RNA was synthesized in the same reaction mixture without biotin-labeled UTP. After in vitro transcription, we used the RNeasy protect mini kit to purify the newly synthesized RNAs for the subsequent experiments. The synthesize RNAs were incubated with BHK-21 cell lysates for 4 h, streptavidin beads were then applied to capture the proteins associated with the biotinylated JEV 3′UTR. Proteins pulled down from cell lysates were separated using 8% SDS-PAGE and silver stained [30]. Four selected gel bands were subjected to in-gel tryptic digestion and the digested peptides were extracted with acetonitrile and dried in a SpeedVac for the LC–MS/MS analysis.

### Reverse-phase LC–MS/MS analysis

The standard procedure of LC–MS/MS was described previously [28]. In brief, the trypsin digested peptides were reconstituted in buffer containing 0.1% formic acid and loaded onto a trap column (Zorbax 300SB-C18, 0.3 × 5 mm; Agilent Technologies, Taiwan) at a flow rate of 0.2 µL/min in HPLC; the peptides were separated on a resolving analytical C18 column (New Objective, Woburn, MA, USA). The peptides were eluted using a linear gradient of 0–10% buffer in 99.9% acetonitrile containing 0.1% formic acid. The liquid chromatograph was then connected to a two-dimensional linear ion trap mass spectrometer (LTQ-Orbitrap, Thermo Fisher, CA, USA). For the MS analysis, we used a data-dependent procedure alternating one mass spectrometry scan and six MS/MS scans for the six most abundant precursor ions.

### Construction of flag-tagged FUBP3 expression plasmid

To generate the 3×Flag-FUBP3 plasmid, we RT-PCR amplified the full-length of FUBP3 from the RNA isolated from BHK-21 cells using the forward primer, 5′-GCGATATCATTCGGCTCCTGAAGCACC-3′, and the reverse primer, 5′-GTTTAAGCCGTAGAATCGTCCATGCG-3′. The PCR product was then treated with *Eco*RV and *Xba*I, and was ligated into the p3×Flag-myc-CMV vector that was previously digested with *Eco*RV and *Xba*I. The constructed plasmid was verified by sequencing. To overexpress the FUBP3 recombinant protein, cells were transfected with 2.5 µg of plasmid DNA using lipofectamine 3000 (Invitrogen) according to the product manual.

### RNA preparation and real-time PCR

RNA extraction was performed as described [31]. Briefly, viral RNA was prepared using QIAamp® viral RNA mini kit (Qiagen, Hilden, Germany). JEV specific single-stranded cDNA was synthesized from 3 µg of cytoplasmic RNA of JEV-infected BHK-21 cells at 2 days post infection. Quantifications of the JE viral RNA were performed by quantitative real time PCR (qPCR) using the orward and reverse primers, GTTTTGGGAGCCTTACTTGT and GCTAAGCATGTTCATCACTA, respectively. The qPCR analyses were performed in duplicate using SYBR green master mix (KAPA) in an ABI 7500 qPCR system.

### Knockdown of FUBP3

We carried out two methods to knockdown the expression levels of FUBP3 in BHK-21 cells. (1) siRNA transfection: FUBP3 siRNA (5′-GUGUCGAGUAGCUAGC-3′) were synthesized by MDBio Inc. Scrambled siRNA was designed and synthesized by MDbio (New Taipei City, Taiwan). The siRNA was transfected into cells using RNAiMAX lipofectamine (Invitrogen) in Opti-MEM reduced serum medium (Invitrogen). The siRNA was incubated with RNAiMAX for 30 min at room temperature prior to transfection, and FUBP3 expression was silenced during 2-day incubation as determined by Western blotting. (2) shRNA transfection: Plasmids containing fubp3 short hairpin RNA (shRNA), pLKO-fubp-3 shRNA (pFubp-3i: GTGTCGAGTAGCTAGC), and a negative control, luciferase shRNA (pNCi: GTACGCGGAATACTTCGA), were obtained from the National RNAi Core Facility, Academia Sinica, Taiwan. BHK-21 cells transfected with pFubp-3 or pNCi were selected with puromycin.

### Western blotting

For Western blotting, an equal amount of cell lysates was denatured for 5 min, separated by 12% SDS-PAGE under reducing conditions, and then electro-transferred to a methanol-activated polyvinylidene difluoride (PVDF) membrane (Bio-Rad Laboratories, Hercules, CA). The membrane was blocked with 5% (wt/vol) nonfat dried milk in PBS-T buffer (20 mM sodium phosphate pH7.4, 137 mM NaCl, and 0.1% Tween 20) at room temperature for 30 min, followed by incubation with mouse anti-FUBP1 and anti-FUBP3 antibodies (Bioworld, Minnesota, USA); rabbit anti-NS5 (Yao-Hong Biotechnology Inc, New Taipei city, Taiwan) and secondary antibodies with an HRP-conjugated goat anti-mouse or anti-rabbit IgG (Sigma, St. Louis, USA), at a 1:10,000 dilution in PBS-T buffer containing 0.5% nonfat milk at room temperature for 1 h. Following three time washes with PBS-T buffer the membrane was developed by ECL (Millipore, MA, USA).

### Viral plaque assay

The standard viral plaque assay was described elsewhere [30]. In brief, a serial tenfold dilutions of the supernatant of JEV infected medium were prepared and infected on the one-layer of BHK-21 cells. After 1-h infection, the medium was removed and cells were washed twice with PBS to remove the unbound viruses. Next, we added 2 mL of RPMI 1640 medium containing 5% FBS and 0.3% seaplaque agarose (Invitrogen, Carlsbad, CA) to each well. The 6 well TC plates were then incubated at 37 °C for at least 4 days, followed by fixing with 2 mL of 10% formaldehyde, and kept for 30 min at room temperature (22–25 °C) before removal of 0.3% agarose. The monolayer cells were stained with crystal violet stain solution (0.5% crystal violet, 1.85% Formalin, 50% EtOH, 0.85% NaCl) (Sigma) and calculated plaque-forming units (pfu/mL) with the virus titer formula, where virus titer equals the number of plaque × (1 mL/0.5 mL) × dilution factor.

### Immunofluorescence and antisera

BHK-21 cells were cultured on glass coverslips for immunofluorescent staining. After infection, the cells were rinsed with PBS and fixed with 4% paraformaldehyde in PBS for 30 min at room temperature. Prior to incubation with antibodies, the cells were permeabilized with 0.1% Triton X-100 in PBS for 30 min and incubated in F1 blocking solution (Biofuture biotech, Taoyuan, Taiwan) for 5 min, followed by incubation sequentially with primary antibodies: mouse anti-FUBP3 protein (Bioworld, Minnesota, USA); rabbit anti-NS5 (Yao-Hong Biotechnology Inc, New Taipei city, Taiwan); rabbit anti-dsRNA (Bioworld, Minnesota, USA) and secondary antibodies: (conjugated with Texas Red) and (conjugated with FITC). After immunostaining, coverslips were mounted on slides in gelvatol medium. Images were acquired using a Zeiss confocal microscope (LSM 510) and processed with Adobe Photoshop software (Adobe, CA).

### Statistical methods

All experiments were repeated at least 3 times. Values are given as means and standard errors of the mean (SEM). Data were analyzed using Graph Pad Prism 4.0 software. Statistical significance was assessed by Student's *t*-test or one-way ANOVA. *p* values less than 0.05 were considered significant.

## Results

### The FUBP3 protein interacted with the JEV 3′UTR

**Fig. 1:**
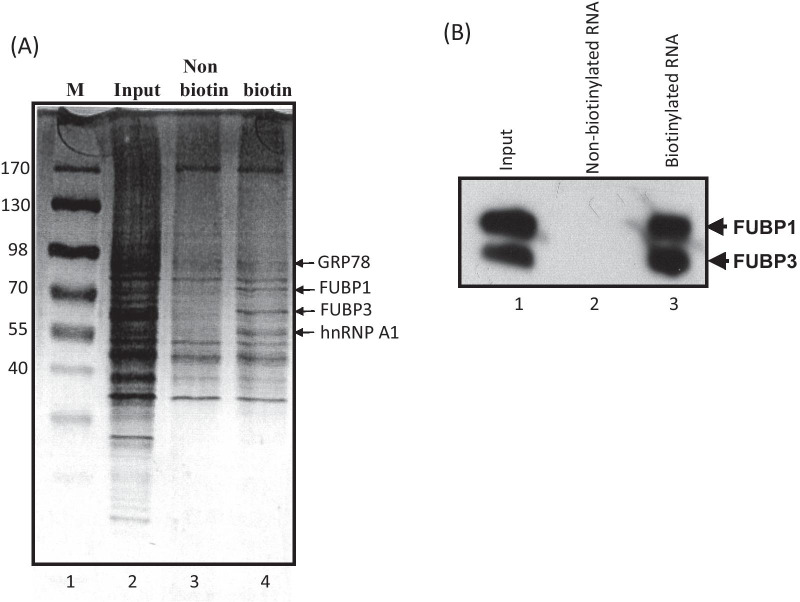
Identification of host proteins associated with JEV 3′UTR. **a** A biotin-labeled JEV 3′UTR was incubated with BHK-21 cell lysates. Streptavidin was then applied to pull down the labeled RNA and associated host proteins. The eluates were subsequently subjected to SDS-PAGE analysis. Lane 1: protein size marker; lane 2: input of BHK-21 cell lysates; lane 3: BHK-21 cell lysates incubated with the non-biotinylated JEV 3′UTR; lane 4: BHK-21 cell lysates incubated with the biotinylated JEV 3′UTR. **b** Western blotting was performed, and both anti-FUBP3 and anti-FUBP1 antibodies were used to detect the presence of these two proteins

To identify host proteins bound to the JEV 3′UTR, the RNA fragment was labeled with biotin and then incubated with BHK-21 cell lysates. After the reaction, streptavidin beads were applied to capture proteins associated with the biotinylated JEV 3′UTR. The biotinylated RNA-associated proteins were pull downed and then separated in SDS-PAGE and observed after silver staining (Fig. 1a). Four bands were observed associating with the biotinylated JEV 3′UTR (Fig. 1a, lane 4) compared to the non-biotinylated JEV 3′UTR (Fig. 1a lane 3). The proteins in these four bands were then subjected to in-gel trypsin digestion and subsequently identified by liquid chromatography tandem-mass spectrometry (LC–MS). For database searches, Mascot Server and Swiss-Prot database were used and further integrated using Scaffold software. The identified proteins were GRP78, FUBP1, FUBP3 and hnRNP A1. Interestingly, GRP78, a molecular chaperone, was among these proteins. GRP78 has been reported to be an important host factor involved in JEV in viral maturation and present in subsequent cellular infections [30]. In addition, we also noted that two far upstream element (FUSE) binding proteins, FUBP1 and FUBP3, were among these proteins. To further confirm the results of RNA pull-down, the biotinylated RNA-associated proteins were eluted for protein electrophoresis. Western blotting was performed to detect the presence of these two proteins using anit-FUBP1 and anti-FUBP3 antibodies (Fig. 1b). FUBP1 has been reported to interact with the JEV 3′UTR and acted as a negative regulator in the JEV infection cycle [29]. Since FUBP3 has been reported to bind RNA and contain helicase activity on the RNA–RNA duplexes [32], we decide to further investigate whether FUBP3 has potential functional role(s) in the JEV infection cycle.

### FUBP3 affects JEV replication

**Fig. 2:**
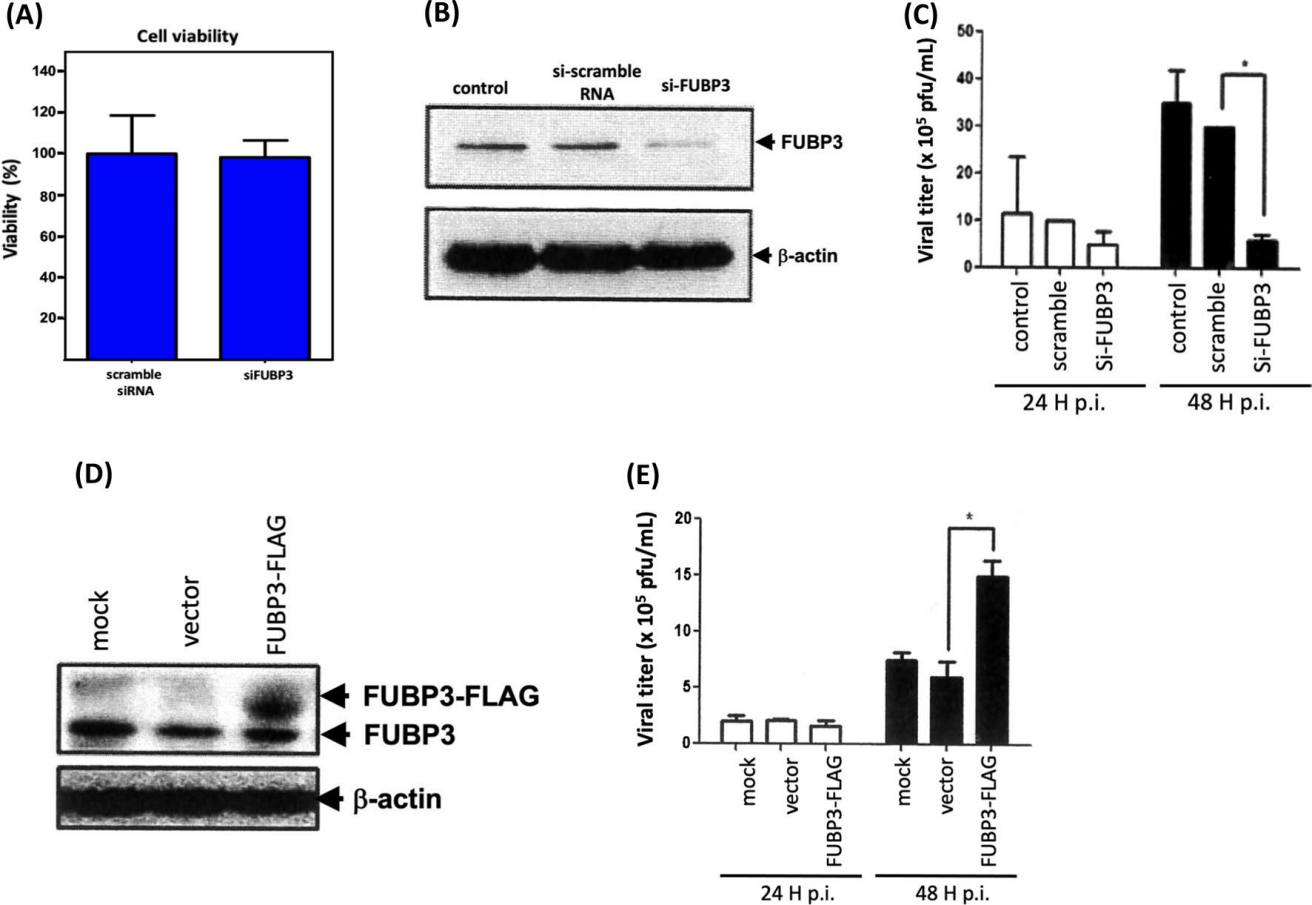
Knockdown of FUBP3 reduces JEV infectivity. **a** BHK-21 cells were transfected with siFUBP3 (5 µM) or si-scramble RNA (negative control), followed by culturing the cells for 24 h prior to the MTT assay. The si-scramble RNA-transfected cells were used as 100% of cell viability. The results shown here were the representative of three independent experiments. **b** BHK-21 cell lysates were harvested and protein expression was analyzed by Western blotting using anti-FUBP3 specific antibody. **c** The si-FUBP3-transfected-BHK-21 cells were then infected with JEV (MOI = 2) for 24 and 48 h, followed by analysis of viral titers using plaque forming assay (*p < 0.05; **p < 0.01). **d** To overexpress the FUBP3, pCMV-8 vector (control) and pCMV-FUBP3-Flag vector (8 µg) were transfected into BHK-21 cells for 24 h. Protein expression was analyzed by Western blotting as described above. **e** FUBP3-overexpressed BHK-21 cells were then infected with JEV (MOI = 2) for 24 and 48 h, followed by analysis of viral titers using plaque forming assay (*p < 0.05; **p < 0.01)

FUBP3 is a protein known to bind RNA and regulate the replication of certain viruses, so we transfected siRNA (siFUBP3) into BHK-21 cells for 24 h and then analyzed the protein expression of FUBP3, followed by infecting the cells with JEV (MOI = 2). First, after transfection with siFUBP3 for 24 h, BHK-21 cells were still viable, as assessed by MTT assay (Fig. 2a). Next, transfection with siFUBP3 resulted in an approximately tenfold decrease in FUBP3 expression in the cells compared to the control (si-scramble) (Fig. 2b). As expected, the viral titers of JEV were significantly reduced by approximately twofold (24 h.p.i) and sixfold (48 h.p.i) compared with the control group (Fig. 2c). As shown above, a decrease in FUBP3 protein was found to cause a significant decrease in JEV viral titers; therefore, we wanted to test whether overexpression of FUBP3 in cells would increase JE viral titer. First, the pCMV-FUBP3-Flag vector was transfected into BHK-21 cells for 24 h. FUBP3 protein expression and JEV viral titer were analyzed. As shown in Fig. 2d, we detected ectopic expression of FUBP3 in pCMV-FUBP3-Flag-transfected BHK-21 cells. JE virus titers increased approximately threefold in cells in the FUBP3-overexpressed cells 48 h after JEV infection compared to the mock and vector control groups (Fig. 2e). From the results, it appears that FUBP3 protein has the ability to regulate JE viral particles production.

### JEV infectivity correlates with the presence of FUBP3

**Fig. 3:**
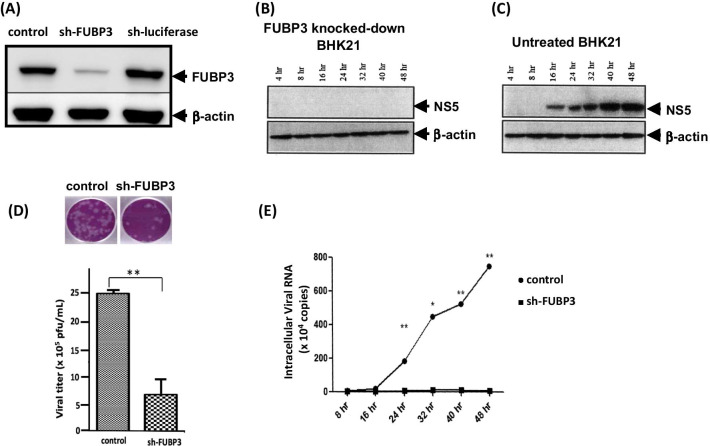
FUBP3 regulated the viral protein translation and viral RNA replication. To establish the stable knocked down FUBP3 cell line, BHK-21 cells were transfected with shFUBP3 plasmid (5 µg) for 72 h, followed by selection with puromycin to obtain the FUBP3 stable knocked-down cells. The expression levels of FUBP3 was detected by Western blotting using anti-FUBP3 specific antibody. A β-actin expression was used as an internal control (**a**). The untreated BHK-21 cells (**c**) and sh-FUBP3-transfected cells (**b**) were then infected with JEV (MOI = 2) for 4, 8, 16, 24, 32, 40, 48 h and protein expression was analyzed by Western blotting using anti-NS specific antibody. A β -actin expression was used as an internal control. **d** Measurement of JE viral titers at 24 h post-infection from untreated and sh-FUBP3-transfected cells were performed by plaque forming assay. **e** The pCMV-FUBP3-Flag vector and pCMV-Flag vector (8 µg) were then transfected in the stable knocked-down FBP3 cells for 24 h, followed by infection with JEV (MOI = 2) for 4, 8, 16, 24, 32, 40, 48 h. The intracellular viral RNA production was then analyzed by RT-qPCR using specific primers as described in the Methods. (*p < 0.05; **p < 0.01; ***p < 0.001)

Since the above results showed that FUBP3 increased JE viral particles production, we next investigated whether FUBP3 could also regulate the viral translation and replication during JEV infection. First, FUBP3 was stably knockdown expressed using the shFUBP3 plasmid, and we detected the decreased expression level of FUBP3 in sh-FUBP3-transfected cells compared to sh-luciferase-transfected and mock BHK-21 cells (Fig. 3a). BHK-21 cells transfected by shFUBP3 remained viable as assessed by the MTT assay in Fig. 2a above (data not shown). Next, cells were then infected with JEV (MOI = 2) and cultured for 4, 8, 16, 24, 32, 40, and 48 h. Viral protein expression was detected by Western blotting using anti-NS5 specific antibody. We found that viral NS5 protein was barely detected in the FUBP3 knocked down cells (Fig. 3b) compared to untreated cells (Fig. 3c), indicating that FUBP3 is an important regulator of JEV protein translation. As expected, the viral titers of JEV were significantly reduced by approximately fivefold (24 h.p.i) compared to the control group (Fig. 3d). Next, in the JEV RNA replication experiment, viral RNA production was analyzed by first stabilizing knockdown cells overexpressed with FUBP3 and then infecting them with JEV (MOI = 2) for 4, 8, 16, 24, 32, 40 and 48 h. As shown in Fig. 3e, when cells with stable knockdown of FUBP3 overexpressed FUBP3, we found a significant increase in viral RNA production over time compared to the control group. The overall results demonstrated that FUBP3 regulates the replication of JEV RNA replication and promoted subsequent viral translation and viral particle production.

### JEV infection redistribute the subcellular localization of FUBP3

**Fig. 4:**
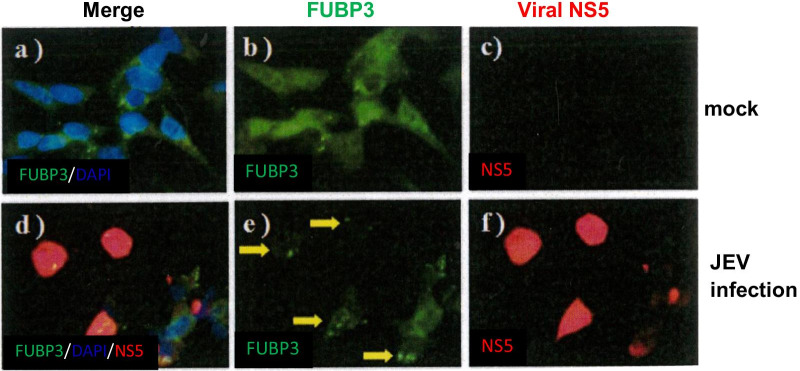
Detection of colocalization of FUBP3 with JEV-NS5 protein in JEV-infected BHK-21 cells. Mock- or JEV-infected BHK-21 cells were harvested at 48 h post-infection and prepared for immunofluorescence analysis stained with antibodies that detect FUBP3 (green) and JEV-NS5 protein (red). Subcellular localization of FUBP3 (panel **b**, **e**) and viral NS5 protein (panel **f**) in mock- and JEV-infected cells. The nucleus was stained with DAPI as shown in the merged image (panel **a**, **c**). Arrows showed the colocalization of FUBP3 with NS protein (panel **e**) were detected in the cytoplasm of JEV-infected cells. The Pearson correlation coefficient (PCC) of images of FITC-FUBP3 and Texas Red-NS5 is 0.9998. Images are representative of three independent experiments that used three independent infections

**Fig. 5:**
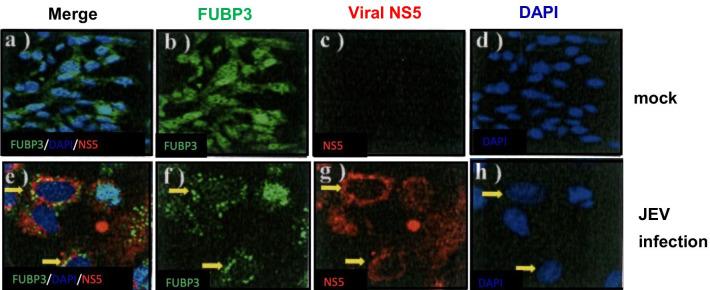
Overexpression of FUBP3 colocalized with viral NS5 protein in JEV-infected BHK-21 cells. Flag-tagged FUBP3 protein that was expressed in BHK-21 cells 24 h prior to JEV infection. The mock- and JEV-infected BHK-21 cells were harvest at 48 h post-infection and co-immunostained with anti-NS5 (red) and anti-FUBP3 (green) antibodies. Subcellular localization of FUBP3 (panel **b**, **f**) and viral NS5 protein (panel **g**) in mock- and JEV-infected cells. The nucleus was stained with DAPI as shown in the merged images (panel **a**/**d**, **e**/**h**). Arrows showed the colocalization of FUBP3 with NS protein (panel **e**) in the cytoplasm of JEV-infected cells. The Pearson correlation coefficient (PCC) of images of FITC-FUBP3 and Texas Red-NS5 is 0.9946. Images are representative of three independent experiments that used three independent infections

It has been shown that FUBP3 protein is present in the cytoplasm and nucleus of cells, and it has been suggested that some viral infections may lead to redistribution of host proteins in cells. In the above experimental results, we know that FUBP3 regulated the replication of JEV, which in turn regulates the subsequent viral translation and viral particles production. Therefore, we wanted to understand whether the endogenous FUBP3 protein redistributes after JEV infection and to confirm the co-localization with the JEV NS5 protein. For this purpose, BHK-21 cells were infected with JEV (MOI = 2) for 48 h and the localization of FUBP3 and JEV NS5 viral proteins was analyzed by confocal microscopy. The results showed that FUBP3 and JEV NS5 viral proteins were co-localized in the cytoplasm (Fig. 4e, f) compared to the control (Fig. 4b, c). The Pearson correlation coefficient measures of images of green fluorescent protein FITC-FUBP3 and Texas Red-NS5 is 0.9998. This implies that after JEV infection, FUBP3 was re-localized in the cytoplasm and co-localized with viral proteins. In addition, we overexpressed the FUBP3 protein in BHK-21 cells prior to JEV infection. As shown in Fig. 5b, the cytoplasm of overexpressed FUBP3 cells had many positive stains for FUBP3 (Fig. 5b), and after infection with JEV, the intracellular distribution of many FUBP3 (Fig. 5f) was affected by JEV and then co-localized with the viral NS5 protein (Fig. 5g), as shown around the viral replication sites (Fig. 5e). Additionally, the Pearson correlation coefficient measures of images of overexpressed FITC-FUBP3 and Texas Red-NS5 is 0.9994, indicating that FUBP3 was co-lcocalized with viral NS5 proteins.

### FUBP3 was recruited to the viral replication complexes of JEV

**Fig. 6:**
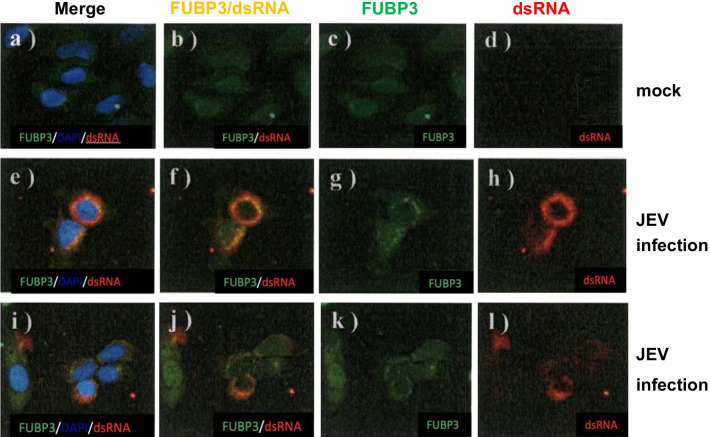
FUBP3 relocated to the JE replication complex. BHK-21 cells were first transfected with the flag-tagged FUBP3 expressing plasmid to overexpress the FUBP3 protein and then proceeded to JEV infection. The mock-and JEV-infected BHK-21 cells were harvest at 48 h post-infection and co-immunostained with anti-dsRNA (red) and anti-FUBP3 (green) antibodies. Panel **a**–**d** are mock groups. Panel **e**–**h** and **i**–**l** are JEV infection groups (Images were taken from three independent experiments, and two representative groups were selected). Positive stained for dsRNA was only observed in the JEV-infected cells (panel **h**, **i**). The nucleus was stained with DAPI as shown in the images (panel **a**, **e**, **f**). The colocalization of FUBP3 with viral dsRNA protein (panel **f**, **j**) was detected in the cytoplasm of JEV-infected cells. The Pearson correlation coefficient (PCC) of images of FITC-FUBP3 and Texas Red-dsRNA is 0.9445. The Images are representative of three independent experiments that used three independent infections

From the above results, it is clear that FUBP3 has a greater effect on JEV replication, as FUBP3 and JEV NS5 protein were found to co-localize in the cytoplasm, and JEV NS5 protein itself is an RNA dependent RNA polymerase responsible for viral replication. Therefore, we speculated whether FUBP3 would be transferred to the JE replication complex to promote viral replication after JEV infection. We infected BHK-21 cells with JEV (MOI = 2) for 48 h and analyzed the localization of FUBP3 and viral double-strand RNA (dsRNA), which is present in the replication complex during replication, by confocal microscopy. The results showed that the co-localization of FUBP3 (Fig. 6g, k) with viral dsRNA (Fig. 6h, i) in the overexpressed FUBP3 cells (merge images: Fig. 6f, j) was more pronounced than in JEV-infected cells alone (control group; Fig. 6k, l). The exogenous FUBP3 was also shown to move into the viral replication complex after JEV infection and to assist in viral replication.

## Discussion

When an RNA virus infects a host cell, it requires the assistance of many host proteins for intracellular replication. The untranslated region of the viral RNA gene itself also plays a key role. In fact, the cyclization of the 3′ untranslated region (3′UTR) is a very important mechanism. In addition to the cyclization of 3′UTR, this untranslated region also binds to some host proteins to promote/inhibit the replication of RNA viruses. In this study, using 3′UTR labeled biotin, we identified four host proteins that bind to the 3′UTR, namely GRP78, FUBP1, FUBP3 and hnRNP A1. GRP78 was reported to be present in the JEV-induced secretion medium and is participated in viral maturation [30]. FUBP1 acts as a defense host factor against JEV by inhibiting viral protein production [29]. Here, we demonstrated that FUBP3 regulated viral RNA replication of JEV and facilitated subsequent viral translation and viral particle production.

Many studies have reported that some host proteins are attracted to bind to the 3′UTR of JEV after infection and affect viral replication or translation. For example, the La protein bound to the SL structure of the JEV 3′UTR, unraveled the secondary structure of viral RNA, and promoted viral replication and translation [33]; DDX5 interacted with the JEV core protein, NS3 and NS5 proteins, moved into the cytoplasm during JEV infection, and bound to the JEV 3′UTR to regulate viral replication and translation [34]. DDX3 interacted with JEV NS3 and NS5 proteins during JEV infection and bound to JEV 3′UTR, deregulating viral translocation and affecting subsequent JEV RNA replication [35]; the polypyrimidine tract-binding protein (PTB) itself functions to assist in the splicing, export and translation of cellular mRNA [36]. In addition to host proteins that bound to the JEV 3′UTR to positively regulate viral replication and translation, some host proteins bound to the JEV 3′UTR to inhibit viral replication, for example, FUBP1 bound to the JEV 5′UTR and 3′UTR to inhibit JEV protein translation, which in turn affected viral replication [29], and zinc-finger antiviral protein (ZAP) bound to the JEV 3′UTR to inhibit viral protein translation and enhance viral RNA degradation. It also destabilized viral RNA by interacting with XRN1 and Rrp46 (EXOSC5), Rrp40 (EXOSC3) and Rrp42 (EXOSC7) [37]. We demonstrated that the FUBP3 protein can positively regulate the translation of JE viral proteins, and after JE virus infects cells, its intracellular location changes and accumulates into granules, which bind to the 3′UTR of the virus gene and affect the translation of the virus, and even further affect the replication of the virus.

FUBP1 and FUBP3 are known to bind to RNA and regulate the replication of certain viruses; for example, FUBP1 was used as an IRES transactivator, competing with FUBP2 for binding to the IRES structure of EV-A71, increasing IRES activity and promoting IRES-mediated translation [38]. As for HCV infection, FUBP1 moved from the cell to the cytoplasm and bound specifically to the poly(U) structure in the poly(U/UC) domain of the HCV 3′UTR, promoting HCV viral replication [39]. In contrast, FUBP1 was reported negatively regulating viral infection, as FUBP1 moved from the nucleus to the cytoplasm during the early stages of JEV infection and bound specifically to the JEV 5′UTR and 3′UTR to inhibit viral replication. The UTR inhibited viral proteins or host proteins to assist viral translocation, further leading to reduced viral replication and affecting viral infection [29]. In contrast to FUBP1 and FUBP2, few reports have mentioned the functional relevance of FUBP3 for RNA viruses. Huang et al. showed that FUBP3 redistributed among subcellular compartments after EV-A71 infection and bound to the 5′UTR of EV-A71 to promote viral replication [28]. In this study, we used RNA pull down assay to identify both FUBP1 and FUBP3 in the JEV-infected BHK-21 cells. FUBP1 has been reported to bind to the UTR of JEV to inhibit JEV protein translation, while our results confirmed that FUBP3 could promote the replication of JEV to increase the viral titers. Previous reports have shown that both FUBP1 and FUBP2, which are also members of the FUBPs family, can bind to the IRES structure of EV-A71 and play the role of ITAFS, positively and negatively regulating the viral protein translocation of EV71, which in turn affects the viral replication [38]. In our study, we demonstrated that the FUBP3 protein can positively regulate the translation of JE viral proteins, and after JE virus infects cells, its intracellular location changes and accumulates into granules. Although both FUBP1 and FUBP3 can bind to the 3′UTR of JEV, the two proteins do not have the same ability to bind, and at the same time, the other host proteins interacting with each of the two proteins are also different. When the cells were infected by JEV, it affected the different levels of expression of various host proteins in the cells, which indirectly interfered with the degree of binding of FUBP1 and FUBP3 to the 3′UTR of JEV, resulting in the different performance of the two proteins binding to the 3′UTR and affecting virus replication. It is possible that the region where the viral 3′UTR is bound competes with FUBP1 and other host factors that inhibit viral replication. In summary, when JEV infects the host cells, FUBP3 in the host cells will accumulate from the original intracellular even distribution into granules, and these granules may be in the same position as the dsRNA of JEV, playing the role of positive regulation of virus replication.

## Conclusion

In the present study, we demonstrated that FUBP3 bound to the 3′untranslated region of JEV and acted as a host factor to promote viral translation, and accompanied the viral replication complexes to accelerate viral RNA replication during the virus life cycle. Knockdown of the FUBP3 protein reduced viral infectivity by disrupting the JEV 3′-UTR-binding host factors. Therefore, inhibition of FUBP3 may be a novel approach to develop effective treatments and prevention strategies for JEV virus infection.

## Data Availability

The datasets used and analyzed during the current study are available from the corresponding author on reasonable request.

## Abbreviations

FUSE: Far upstream element
FUBP: Far upstream element-binding protein
JEV: Japanese encephalitis virus
UTR: Untranslated region
UAR: Upstream of AUG region
DENV: Dengue virus
WNV: West Nile virus
MOI: Multiplicity of infection
